# Ambient Air Pollution and Cardiometabolic Health in the Tehran Lipid and Glucose Study: A Scoping Review of Two Decades of Evidence

**DOI:** 10.5812/ijem-166630

**Published:** 2025-10-30

**Authors:** Amir Hossein Hasanpour, Mohammad Javad Mansourzadeh, Alireza Khajavi, Fereidoun Azizi, Farzad Hadaegh

**Affiliations:** 1 Prevention of Metabolic Disorders Research Center, Research Institute for Metabolic and Obesity Disorders, Research Institute for Endocrine Sciences, Shahid Beheshti University of Medical Sciences, Tehran, Iran; 2 Endocrine Research Center, Research Institute for Endocrine Disorders, Research Institute for Endocrine Sciences, Shahid Beheshti University of Medical Sciences, Tehran, Iran

**Keywords:** Ambient Air Pollution, Particulate Matter, Environmental Exposure, Cardiometabolic Risk Factors, Scoping Review

## Abstract

**Context:**

This scoping review investigates the association between ambient air pollution and cardiometabolic outcomes using data from the Tehran Lipid and Glucose Study (TLGS), a population-based cohort initiated in 1999.

**Evidence Acquisition:**

Five TLGS studies were included, each examining associations between ambient air pollutants and cardiometabolic outcomes such as hypertension (HTN), dyslipidemia, diabetes, cardiovascular morbidity, and mortality. Due to overlapping populations but differing outcome measures across studies, a meta-analysis was not feasible. Instead, a narrative synthesis was conducted, with results organized into a comparative matrix to facilitate cross-study evaluation.

**Results:**

The findings reveal heterogeneous effects of ambient air pollutants on cardiometabolic health across both short- and long-term pathways. Short-term exposures to nitrogen dioxide (NO₂) and particulate matter ≤ 10 µm in diameter (PM₁₀) were linked to higher systolic blood pressure (SBP), while sulfur dioxide (SO₂) was associated with elevated diastolic pressure. For lipid parameters, carbon monoxide (CO) and SO₂ corresponded with higher total cholesterol (TC), triglycerides (TG), low-density lipoprotein cholesterol (LDL-C), and non-high-density lipoprotein cholesterol (non-HDL-C) levels. Cardiovascular outcomes also worsened, as increases in the Air Quality Index (AQI), PM₁₀, SO₂, and CO were associated with higher risks of cardiovascular disease (CVD) hospitalization and mortality. Long-term exposures to ozone (O₃), PM₁₀, and SO₂ predicted incident HTN (strongest for PM₁₀), while CO was associated with elevated TC, TG, and adverse dyslipidemia phenotypes but not high LDL-C. Sulfur dioxide, O₃, and PM₁₀ also increased risks of dysglycemia, though no consistent associations with type 2 diabetes incidence or long-term mortality were observed.

**Conclusions:**

This review underscores the substantial influence of ambient air pollution on metabolic and cardiovascular health in Tehran. Short-term exposure to pollutants such as PM₁₀, SO₂, and CO is associated, either immediately or with lagged effects, with increased blood pressure, adverse lipid changes, and heightened cardiovascular risk, while long-term exposure to PM₁₀ and SO₂ exacerbates HTN and impairs glucose metabolism. These findings highlight the need for stricter air quality regulations and further investigation into the cumulative, long-term effects of air pollution in the metropolitan city of Tehran.

## 1. Context

Ambient air pollution has significantly increased over the past two decades and, in 2021, was identified as the leading environmental risk factor for global mortality, particularly in urban centers. It is responsible for over eight million deaths annually, with more than 90% occurring in low- and middle-income countries (1-3). According to the Global Burden of Disease (GBD) study, particulate matter (PM₁₀) is the largest environmental contributor to disease burden, accounting for 8.0% of total disability-adjusted life years (DALYs), surpassing traditional metabolic risk factors like high systolic blood pressure (SBP) (4).

Iran, particularly Tehran, is experiencing a rising prevalence of non-communicable diseases, including hypertension (HTN), dyslipidemia, and type 2 diabetes (5, 6). In Tehran, air pollution is a growing concern, with vehicular traffic, industrial emissions, and geographical features contributing to high levels of ambient pollutants. These factors are linked to the increasing burden of cardiometabolic diseases, including myocardial infarction (MI), stroke, and premature mortality (7-10).

The Tehran Lipid and Glucose Study (TLGS), a population-based cohort initiated in 1999, has provided extensive data on risk factors for non-communicable diseases (11, 12). By integrating TLGS data with regional air pollution monitoring, the study has enabled detailed assessments of ambient pollutants' effects on various cardiometabolic outcomes (6, 10).

Recent TLGS studies have highlighted associations between PM₁₀, sulfur dioxide (SO₂), nitrogen dioxide (NO₂), ozone (O₃), and carbon monoxide (CO) with increased risks of HTN (10), dysglycemia (6), dyslipidemia (13), cardiovascular events and mortality (14), and cardiovascular hospitalization (15). However, existing research is fragmented across multiple publications with varying methodologies and exposure assessments, hindering comprehensive interpretation and the development of effective public health strategies. A scoping review is ideal for mapping existing evidence, clarifying research boundaries, and identifying gaps in knowledge without the limitations of meta-analytic homogeneity (16-18).

This scoping review aims to assess TLGS literature on ambient air pollution and cardiometabolic outcomes. The review will address four key questions: (1) Which cardiometabolic outcomes have been studied? (2) What pollutants and exposure windows have been examined, and what methods were used? (3) What are the main findings? (4) What gaps remain, and what future research directions are needed? The objective is to map available evidence and identify research priorities, rather than derive pooled quantitative estimates.

## 2. Evidence Acquisition

### 2.1. Review Design

This scoping review was conducted in accordance with the PRISMA-ScR (Preferred Reporting Items for Systematic Reviews and Meta-Analyses Extension for Scoping Reviews) guidelines (19). Its primary objective was to map the existing evidence on the association between ambient air pollution and cardiometabolic health outcomes, with a specific focus on studies conducted within the TLGS cohort. The TLGS is a large-scale, longitudinal study that provides robust data on environmental exposures and their relationships with a wide range of health outcomes. The details of the cohort characteristics are presented elsewhere (11, 12).

### 2.2. Eligibility Criteria

Studies were eligible for inclusion if they were conducted within the TLGS cohort, assessed the impact of ambient air pollution on cardiometabolic outcomes such as HTN, dyslipidemia, diabetes, cardiovascular events, and all-cause mortality, and adopted a cohort study design to evaluate associations between exposure and outcome.

### 2.3. Information Sources

All data for this review were obtained exclusively from studies conducted within the TLGS cohort. External databases and grey literature were not searched, as all pertinent evidence on the relationship between ambient air pollution and cardiometabolic outcomes was already available from TLGS-based research.

### 2.4. Selection of Sources of Evidence

The selection process began with the screening of all TLGS publications according to the predefined eligibility criteria. Studies that specifically examined the association between ambient air pollution and cardiometabolic outcomes were retained. In total, five studies met the eligibility criteria and were included in the review. Given our objective to exclusively map and synthesize the evidence generated from the well-defined TLGS cohort, a broader database search was deemed outside the scope of this particular review, therefore no further studies were considered.

### 2.5. Data Charting

Data extraction was conducted manually by one author, and the results were independently verified by a second author to ensure accuracy and consistency. Extracted data included bibliographic details, study design and analytical approach, TLGS phases and calendar period, sample size and participant demographics, pollutants assessed, exposure metrics and sources, cardiometabolic outcomes, statistical models, main results, adjusted confounders, and author-reported limitations.

### 2.6. Synthesis of Results

Meta-analysis was not feasible due to heterogeneity in outcomes, exposure periods, and study populations. Instead, a narrative synthesis was performed. The extracted data were systematically organized in a comparative matrix to enable cross-study evaluation of pollutant types and cardiometabolic outcomes, thereby facilitating coherent interpretation despite methodological variability across studies.

### 2.7. Assessment of Methodological Quality

No formal critical appraisal was conducted, as all eligible TLGS publications were included. However, study designs, statistical models, outcomes, and reported limitations were comprehensively documented and considered during synthesis.

## 3. Results

### 3.1. Study Characteristics

**Figure 1.**
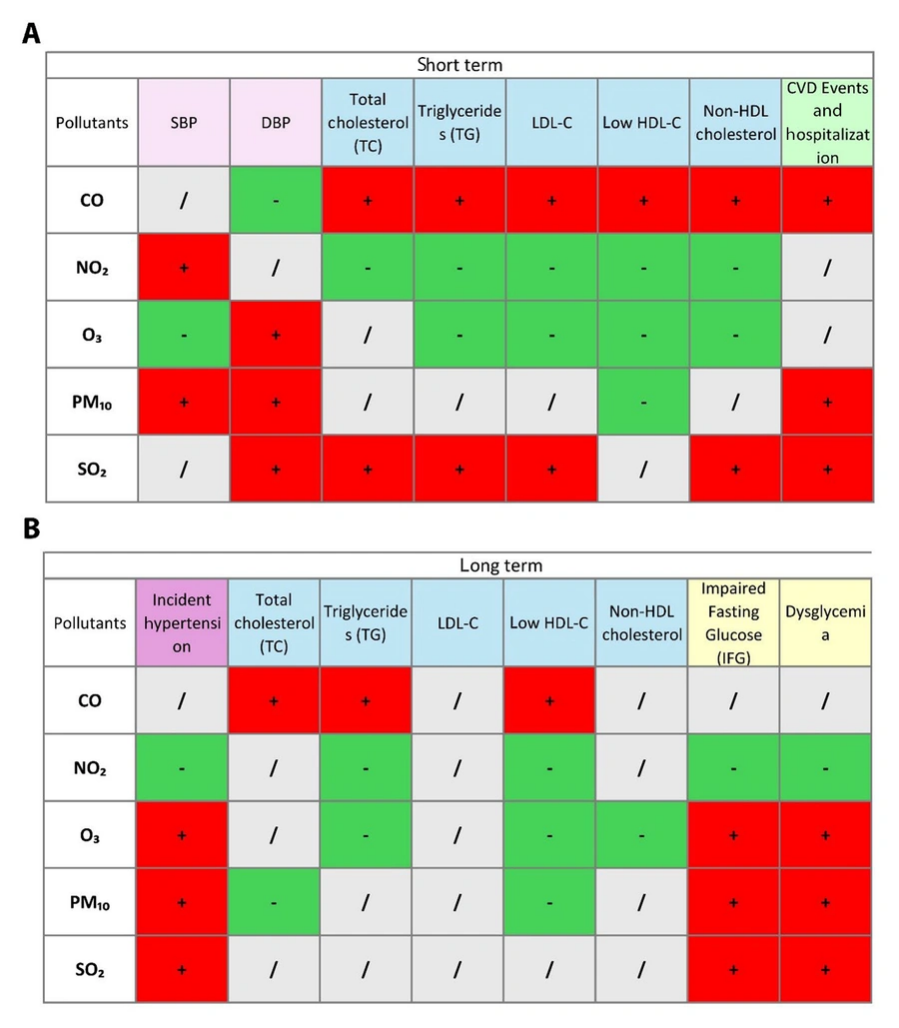
Matrix of associations between ambient air pollutants and cardiometabolic health outcomes in the short-term and long-term; short-term (A), and long-term (B) associations between ambient air pollutants (CO, NO₂, O₃, PM₁₀, and SO₂) and various cardiometabolic health outcomes, including blood pressure (SBP, DBP), lipid profiles (total cholesterol, triglycerides, LDL-C, HDL-C, non-HDL cholesterol), and cardiovascular events and hospitalization. Positive (+) and negative (-) associations are indicated in green and red, respectively. Neutral/no association is marked in gray (abbreviations: CO, carbon monoxide; NO₂, nitrogen dioxide; O₃, ozone; PM₁₀, particulate matter ≤ 10 µm in diameter; SO₂, sulfur dioxide; SBP, systolic blood pressure; DBP, diastolic blood pressure; LDL-C, low-density lipoprotein cholesterol; HDL-C, high-density lipoprotein cholesterol; non-HDL, non-high-density lipoprotein).

**Figure 2.**
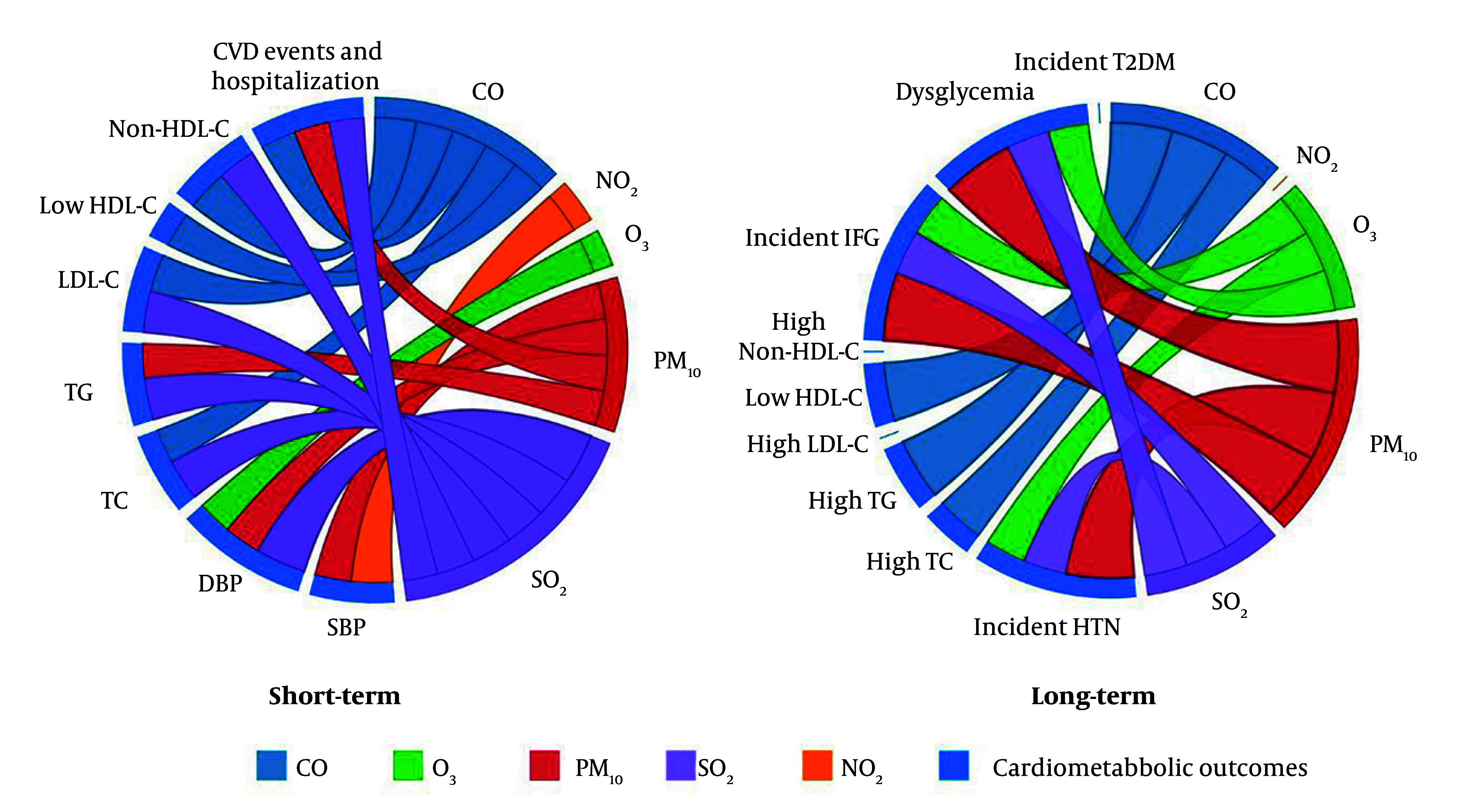
Chord diagram of positive associations between ambient air pollutants and cardiometabolic health outcomes in the short-term and long-term; short-term and long-term associations between ambient air pollutants (CO, NO₂, O₃, PM₁₀, and SO₂) and various cardiometabolic health outcomes, including blood pressure (SBP, DBP), lipid profiles (total cholesterol, triglycerides, LDL-C, HDL-C, non-HDL cholesterol), and cardiovascular events & hospitalization (abbreviations: CO, carbon monoxide; NO₂, nitrogen dioxide; O₃, ozone; PM₁₀, particulate matter ≤ 10 µm in diameter; SO₂, sulfur dioxide; SBP, systolic blood pressure; DBP, diastolic blood pressure; LDL-C, low-density lipoprotein cholesterol; HDL-C, high-density lipoprotein cholesterol; non-HDL, non-high-density lipoprotein).

Five TLGS papers (2019 - 2025) met inclusion criteria. Two cardiovascular-event studies analyze TLGS Phase 1 recruits from 1999 (15, 16), while three risk-factor studies start in Phase 2 from 2001 and follow participants through Phase 6. Sample sizes range from roughly 3,500 to 9,700 adults. Four papers model short-term (0 - 14-day) lags, three examine long-term (1- to 3-year) averages, and two cover both. All rely on identical city-wide metrics for PM₁₀, SO₂, NO₂, O₃, and CO. Outcomes span continuous blood pressure and incident HTN, longitudinal serum lipids and dyslipidemia, incident dysglycemia/diabetes, adjudicated fatal and non-fatal cardiovascular events and hospitalizations, and all-cause/cause-specific mortality. Study characteristics are summarized in Table 1. The distribution of air pollutants during 1999 - 2018 (15) is shown in Table 2, and associations between pollutants and outcomes are presented in a matrix in Figure 1. Moreover, a chord diagram of positive short- and long-term associations between pollutants and cardiometabolic outcomes are presented in Figure 2A and 2B, respectively.

**Table 1.** Basic Characteristics of the 5 Included Studies

#	Bibliographic Details/Study Design	Study Design and Analytical Approach	TLGS Cycle(s) and Calendar Period	Sample and Demographics	Pollutants (Lags/Averaging Windows)	Exposure Metric and Source	Cardiometabolic Outcomes	Key Results (Effect Size ± 95% CI)	Confounders Adjusted	Author-Reported Limitations
**1**	Khajavi A. et al., 2019. Sci Total Environ 661:243-250 – Iran – Prospective cohort time-series (14)	Daily cohort time-series; DLNM; zero-inflated quasi-Poisson	Cycles 1-5; 1999 - 2014 follow-up	9 731 adults ≥ 30 y (4 409 men)	Composite AQI (primary); single-pollutant sensitivity (PM₁₀); lags 0-7 d (peaks at 2 & 6 d)	23 fixed monitors; city-wide daily mean (TehranAQI)	Daily fatal+non-fatal CVD events; all-cause deaths	CVD: AQI 180 vs. 50, lag 2 d RR 1.94 (1.02 - 3.67); lag 6 d RR 2.06 (1.09 - 3.88). Death: AQI 180, lag 1 d RR 2.40 (1.00 - 5.59)	Natural splines of temperature; Day-of-week; Long-term time trend; Population offset	Central-site exposure; Zero-inflated counts; Limited events; Ecological inference
**2**	Khajavi A. et al., 2021. Int J Hyg Environ Health 234:113719 – Iran – Longitudinal cohort (10)	Mixed-effects transition model (SBP/DBP, lags 0-14 d); Weibull proportional-hazards (interval-censored) for incident HTN	Cycles 2 - 6; 2001 - 2018	4 580 non-hypertensive adults 20 - 69 y (41.6 % men)	PM₁₀, SO₂, NO₂, O₃, CO; lags 0-14 d and 1-, 2-, 3-y means	23-station daily averages (Tehran AQI)	Continuous SBP/DBP; Incident HTN	3-y PM₁₀ ↑ 10 µg/m³ → HR 1.96 (1.48 - 2.62) incident HTN	Age, sex, BMI, WC, diabetes, anti-HTN drug use, ever-smoker, temperature	Central monitor; No PM₂.₅; Urban-only generalizability
**3**	Khajavi A. et al., 2025. Int J Hyg Environ Health 266:114573 – Iran – Prospective cohort time-series (15)	Daily counts; DLNM; zero-inflated quasi-Poisson	Cycles 1 - 6 (subset 50 - 70 y); 1999 - 2018	3 454 adults 50 - 70 y (54% women)	CO, PM₁₀, SO₂, NO₂, O₃; single-day and distributed lags 0 - 7 d	Citywide mean of 23 monitors	CVD hospitalizations (MI, stroke, HF, unstable angina, PAD+other specified)	CO lag 0 (IQR ≈ 5 mg/m³): RR 1.92 (1.65 -2.23); PM₁₀ lag 1 (IQR ≈ 12 µg/m³): RR 1.12 (1.01 - 1.24); SO₂ lag 2: RR 1.06 (1.04 - 1.07)	Temperature, day-of-week, seasonality, long-term trend	Central-site exposure; Age-restricted cohort; Missing residential data; Urban-only focus
**4**	Tamehri Zadeh S. S. et al., 2022. Environ Sci Pollut Res 30:3213-3221 – Iran – Prospective cohort (6)	Weibull PH (interval-censored) for long-term dysglycaemia; 1-, 2-, 3-y averages	Cycles 2-6; 2001 - 2018	4 254 normoglycaemic adults 20 - 69 y (40.4% men)	PM₁₀, SO₂, O₃, NO₂, CO; annual means (1 - 3 y)	Mean of 23 monitors; No PM₂.₅ data	Incident dysglycaemia, IFG, type 2 diabetes	PM₁₀ 3 - y ↑ 10 µg/m³ → HR 2.20 (1.67 - 2.89) incident dysglycaemia	Age, sex, BMI, WC, family history diabetes, HTN, ever-smoker, temperature	Central monitor; Mobility-related exposure error; Lack of rural data; No PM₂.₅
**5**	Tamehri Zadeh S. S. et al., 2023. Atmos Environ 306:119796 – Iran – Longitudinal cohort (13)	LME (short-term 1 - 14 d); Weibull PH for incident dyslipidaemia	Cycles 2 - 6; 2001 - 2018	5 821 adults 20 - 69 y (58.2% women)	PM₁₀, SO₂, O₃, NO₂, CO; Moving av. 1 - 14 d; 1 - 3 y means	23-station average (Tehran AQI)	Continuous lipids (TC, TG, LDL-C, HDL-C, non-HDL-C); Incident high TC/TG, low HDL-C, etc.	CO 3-y ↑ 1 mg/m³ → HR 1.80 (1.50 - 2.30) high TG; HR 1.79 (1.30 - 2.52) low HDL-C	Sex, age, BMI, PA, smoking, prevalent CVD, temperature	Central monitor; Healthier-participant bias; No PM₂.₅; Urban context only

Abbreviations: AQI, Air Quality Index; BMI, Body Mass Index; CI, confidence interval; CO, carbon monoxide; CVD, cardiovascular disease; DBP, diastolic blood pressure; DLNM, distributed-lag non-linear model; HF, heart failure; HR, hazard ratio; HTN, hypertension; IFG, impaired fasting glucose; IQR, interquartile range; LDL-C, low-density-lipoprotein cholesterol; LME, linear mixed-effects; MI, myocardial infarction; NO₂, nitrogen dioxide; O₃, Ozone; PAD, peripheral artery disease; PA, physical activity; PH, proportional hazards; PM₁₀, particulate matter ≤ 10 µm; PM₂․₅, particulate matter ≤ 2.5 µm; RR, relative risk; SBP, systolic blood pressure; SO₂, sulfur dioxide; TC, total cholesterol; TG, triglycerides; WC, waist circumference; non-HDL-C, non-high-density lipoprotein cholesterol.

**Table 2.** The Distribution of Temperature and Air Pollutants During the Study Period 1999 - 2018 (15) ^a^

	PM_10_	SO_2_	NO_2_	CO	O_3_
**WHO thresholds**	45	40	25	4	100
**Mean ± SD**	61.4 ± 20.0	31.7 ± 14.5	44.6 ± 22.2	3.2 ± 1.6	36.9 ± 20.1
**Min-Max**	5 - 281	3 - 113	6 - 110	0.8 - 30	2 - 160
**IQR**	22	19	38	1.7	25

Abbreviations: WHO, World Health Organization; SD, standard deviation; IQR, interquartile range.

^a^ Values are expresses as percentage or mean ± SD (µg/m³).

### 3.2. Blood-Pressure Outcomes

#### 3.2.1. Systolic Blood Pressure

Short-term exposure to CO did not affect SBP across any of the 14-day lag periods. Exposure to NO₂ was associated with increased SBP, with a significant positive association observed throughout the 14-day lag period. Ozone exposure was linked to a reduction in SBP across the full lag window. Particulate matter was associated with elevated SBP within the 0 - 3-day lag range. Sulfur dioxide did not demonstrate a statistically significant association with SBP across any lag periods.

#### 3.2.2. Diastolic Blood Pressure

Short-term exposure to CO was significantly associated with reduced diastolic blood pressure (DBP) across all 14-day lag periods. Nitrogen dioxide exposure had no significant effect on DBP. Ozone exposure produced a slight, statistically non-significant increase in DBP. Particulate matter exposure was associated with increased DBP during lags 0 - 3 days and again at 12 - 14 days. Sulfur dioxide exhibited the strongest and most consistent effect on DBP, showing statistically significant increases throughout all 14-day lag periods.

#### 3.2.3. Incident Hypertension (Long-Term Outcome)

Incident HTN was evaluated as the sole long-term blood pressure outcome using interval-censored Weibull survival models over 1-, 2-, and 3-year average pollutant exposures. Long-term exposure to CO had no statistically significant association with incident HTN. Nitrogen dioxide was associated with a significantly lower risk of developing HTN over the 2- and 3-year exposure windows. In contrast, O₃ was associated with an increased risk of incident HTN in all three exposure windows. Similarly, PM₁₀ was consistently associated with an increased risk of HTN, with the strongest effect observed for 3-year exposure [hazard ratio (HR): 1.96; 95% confidence interval (CI): 1.48 - 2.62]. Sulfur dioxide also showed a positive association with incident HTN consistently across all three time-spans (10).

### 3.3. Lipid-Profile Outcomes

#### 3.3.1. Total Cholesterol

Short-term exposure produced clear pollutant-specific patterns. Carbon monoxide and SO₂ raised total cholesterol (TC) across the 14-day moving-average window, whereas NO₂ produced a significant fall in TC over the same lags. Ozone showed no material association with TC, and PM₁₀ had at most a minimal, non-significant effect.

Long-term analyses, based on 1-, 2-, and 3-year mean concentrations, identified CO as the only pollutant that increased the hazard of developing high TC; the risk rose progressively with longer exposure. Particulate matter was linked to a lower incidence of high TC after three years, while NO₂, O₃, and SO₂ showed no significant long-term relation with high TC.

#### 3.3.2. Triglycerides

In the short term, both CO and SO₂ consistently elevated triglycerides (TG), the effect size intensifying as the 14-day moving average lengthened. By contrast, NO₂ and O₃ were each associated with lower TG across the same lag structure, and PM₁₀ exerted no discernible influence.

For long-term risk of high TG, CO again emerged as an adverse factor, showing a significant positive association in all three exposure windows. Nitrogen dioxide and O₃ were opposite, each conferring a lower risk of high TG over two- and three-year averages, whereas PM₁₀ and SO₂ did not reach significance after multivariable adjustment.

#### 3.3.3. Low-Density Lipoprotein Cholesterol

Short-term increments in CO and SO₂ increased low-density lipoprotein cholesterol (LDL-C), while NO₂ and O₃ produced statistically significant reductions. Particulate matter had no material impact on LDL-C.

In the long-term interval-censored Weibull survival models, none of the five pollutants showed a significant association with incident high LDL-C after adjustment for demographic and lifestyle covariates.

#### 3.3.4. High-Density Lipoprotein Cholesterol

Short-term exposure to CO lowered high-density lipoprotein cholesterol (HDL-C) throughout the 14-day lag period. Nitrogen dioxide and O₃ each raised HDL-C, and PM₁₀ produced a small but statistically significant increase. Sulfur dioxide showed no consistent effect on HDL-C.

Long-term results paralleled the short-term findings: Carbon monoxide increased the risk of developing low HDL-C, whereas NO₂ and O₃ were associated with lower risk. Particulate matter also showed inverse association, demonstrating a significant reduction in incident low HDL-C after one and two years of exposure, while SO₂ remained neutral.

#### 3.3.5. Non-high-Density Lipoprotein Cholesterol

For non-HDL cholesterol, the short-term pattern resembled that of TC: Carbon monoxide and SO₂ elevated non-HDL levels, NO₂ and O₃ reduced them, and PM₁₀ showed no meaningful effect.

In long-term analysis, a single significant finding emerged: three-year exposure to PM₁₀ was associated with a lower incidence of high non-HDL cholesterol. Carbon monoxide, NO₂, O₃, and SO₂ were not materially related to long-term risk of high non-HDL cholesterol (13).

### 3.4. Glucose-Metabolism Outcomes

#### 3.4.1. Impaired Fasting Glucose

Short-term effects of ambient pollutants on fasting glucose were not evaluated in the TLGS analyses; therefore, only long-term findings are reported. In the fully-adjusted Weibull models, a clear pollutant-specific pattern emerged. Mean annual exposures to SO₂ and O₃ raised the risk of incident impaired fasting glucose (IFG) across all three exposure windows, and PM₁₀ raised the risk at one- and three-year averages (the two-year window was not significant as CIs crossed 1). Nitrogen dioxide showed the opposite behavior, reducing IFG risk in every time window. Carbon monoxide had no measurable influence on IFG. The strongest adverse effect was seen for PM₁₀ after three years of exposure, HR 2.08 (95% CI 1.55 - 2.80), whereas the largest protective effect was recorded for NO₂ after three years, HR 0.82 (0.72 - 0.94).

#### 3.4.2. Dysglycemia

The overall incidence of dysglycemia was significantly influenced by long-term exposure to SO₂, O₃, and PM₁₀. These pollutants were linked to an increased risk of dysglycemia, with PM₁₀ showing the highest risk, notably after 3 years of exposure, where the HR reached 2.20 (95% CI 1.67 - 2.89). Similar to IFG, NO₂ exposure was inversely associated with the development of dysglycemia, particularly in the first year of exposure, where a modest decrease in risk was observed (HR 0.89, 95% CI 0.80 - 0.98), and no significant associations were detected for CO.

#### 3.4.3. Type 2 Diabetes Mellitus

Most pollutants demonstrated no significant long-term association with incident diabetes after multivariable adjustment. Carbon monoxide, SO₂, O₃, and PM₁₀ showed no significant associations in any exposure window after multivariable adjustment. Nitrogen dioxide differed from the other pollutants. A one-year increase in NO₂ was linked to a modest but significant reduction in diabetes risk (HR 0.89, 95% CI 0.80 - 0.98), while the two- and three-year averages did not reach statistical significance. Thus, apart from this isolated inverse association for NO₂, long-term ambient pollution was not a decisive determinant of incident type 2 diabetes in the TLGS (6).

### 3.5. Cardiovascular Morbidity and Mortality

#### 3.5.1. Cardiovascular Diseases and Mortality

In the Khajavi, et al. 2019 study, the focus was on the relationship between the Air Quality Index (AQI) and cardiovascular mortality. The AQI is a numerical scale that represents the overall air quality based on the maximum scaled values of six key pollutants, including PM₁₀, NO₂, SO₂, CO, and O₃. The higher the AQI value, the more polluted the air. The results indicated that high AQI values were linked to an increased risk of cardiovascular death. However, the study did not find significant long-term effects of air pollution on mortality across the entire sample, but immediate exposure to higher AQI values did lead to higher mortality risks. This suggests that short-term exposure to high pollution levels, as measured by the AQI, is particularly harmful in terms of cardiovascular mortality.

The subgroup analysis in this study revealed notable differences in the impact of air pollution on cardiovascular disease (CVD) and mortality across age groups. For individuals under 60 years old, significant risks for CVD were observed at higher levels of air pollution (AQI = 180) with a lag of 2 and 6 days, where relative risks (RR) of 1.94 and 2.06 were reported, respectively. In contrast, no significant effect on CVD was found for individuals aged 60 and above. Regarding mortality, significant effects were also observed in the under-60 group, with the highest risk at AQI = 180 and lag 1 day (RR = 3.29). For the 60+ years subgroup, the highest risk for death due to air pollution occurred at AQI = 180 and lag 7 days, with an RR of 2.16. These findings highlight that younger individuals were more immediately and significantly affected by air pollution in terms of both CVD and mortality, whereas elderly individuals displayed a delayed response, with risks peaking at longer lags (14).

#### 3.5.2. Cardiovascular Disease Events and Hospitalization

The 2025 study by Khajavi, et al. reported that short-term exposure to ambient air pollutants, particularly PM₁₀, SO₂, and CO, was significantly associated with an increased risk of CVD hospitalization. Elevated PM₁₀ concentrations, especially those exceeding 70 µg/m³, were linked to a heightened risk of hospitalization, with the strongest effect observed within one day of exposure. Similarly, SO₂ levels as low as 24 µg/m³ were associated with increased hospitalization risk, and this association persisted at higher concentrations, particularly at a six-day lag. Carbon monoxide exposure was also positively associated with hospitalization risk, with the greatest effect occurring shortly after exposure, especially at concentrations above 3 mg/m³. In contrast, NO₂ exposure above 68 µg/m³ was associated with a reduced risk of hospitalization, although this inverse association was not statistically significant across all lag periods. No significant association was observed for O₃ overall; however, a concentration of 50 µg/m³ corresponded to a 20% increase in hospitalization risk among individuals with chronic kidney disease (CKD) or metabolic syndrome (MetS).

The subgroup analysis reveals that pollutant effects vary significantly across different groups based on sex, CKD status, and MetS status. Particulate matter shows significant effects primarily in women, CKD, and MetS individuals, with increasing risk at higher concentrations. Ozone has no significant effect across any subgroup. Sulfur dioxide consistently demonstrates significant effects in all groups, peaking at 49-50 μg/m³. Carbon monoxide exhibits notable effects at higher concentrations (5 mg/m³) across all subgroups, with lagged effects observed in CKD and MetS individuals, particularly at 5-6 days for MetS. Nitrogen dioxide is associated with reduced risk at higher concentrations in all groups, with the lowest risk recorded at 86 μg/m³. Lagged effects are primarily seen for CO, with significant lags in non-CKD and MetS individuals, while other pollutants, such as PM₁₀, SO₂, and NO₂, show no lag effects. These findings highlight the differential impacts of air pollutants depending on demographic and health conditions (15).

## 4. Discussion

ehran, the most densely populated metropolitan area in Iran, has been a focal point of research given its substantial burden of cardiometabolic diseases [20 - 24]. Building on previous evidence, this study highlights that a considerable share of this burden is attributable to ambient air pollution. In particular, exposure to pollutants such as CO, SO₂, and PM₁₀ appears to exacerbate both risk factors and outcomes related to cardiometabolic health.

### 4.1. Air Pollution and Blood Pressure

In Tehran, HTN represents a major public health challenge, affecting an estimated 40% of the population, with an additional 12% classified as pre-hypertensive (20-22). Despite a relatively high awareness rate of 70%, only 50% of affected individuals seek treatment, and merely 40% achieve their treatment goals (20). Findings from the TLGS are consistent with prior Iranian evidence, showing that exposure to PM₁₀, SO₂, and O₃ is associated with incident HTN. In parallel, a 2017 national study across several Iranian cities reported that PM₁₀ concentrations were significantly associated with 55% greater risk for incident HTN (23). Another study highlighted a similar relationship between particulate matter ≤ 2.5 µm (PM₂.₅), O₃, and HTN, while no significant association was found with NO₂ exposure (24). Complementary evidence from the CASPIAN-III study in adolescents revealed a significant positive correlation between AQI and SBP (25).

Similar patterns have been observed internationally. A 2016 Systematic Review and Meta-Analysis of 17 studies examining the associations of exposure to air pollutants with HTN, reported that long-term exposure to NO₂ and PM₁₀ had significant associations with HTN (26). Moreover, a newly published umbrella review of systematic reviews and meta-analyses further demonstrated that short-term exposure to PM₂.₅ is associated with increased systolic and diastolic BP, as well as with long-term incidence of HTN, while PM₁₀ exposure was only linked specifically to elevated DBP in both the short- and long-term exposure (27). Also, a recent Danish cohort study of 32,851 participants found an increase in SBP linked to NO₂ exposure, with no association for DBP, reflecting trends seen in the TLGS (28). In line with these observations, the recently updated American Heart Association (AHA) guideline for HTN management identifies air pollution, in particular fine particulate matter (PM₂.₅), as a key non-dietary risk factor for HTN (29, 30). However, our study extends this perspective by highlighting that coarse particulate matter (PM₁₀) also plays a crucial, yet often underappreciated, role in the development of HTN.

### 4.2. Air Pollution and Serum Lipids

Recent national surveillance studies report that nearly 80% of Iranian adults have at least one lipid abnormality, with low HDL-C and hyper TG being the most prevalent. Although high TC and LDL-C showed a declining trend from 2016 to 2021, the overall prevalence of dyslipidemia remained unchanged due to persistent low HDL-C and rising TG levels (31, 32). Ambient air pollution is a recognized risk factor for adverse lipid profiles and cardiovascular events (33). In TLGS, short-term exposure to CO and SO₂ was linked to unfavorable lipid changes, whereas long-term exposure showed minimal associations. These findings align with the CASPIAN-III adolescent cohort, which reported higher TC and TG levels associated with AQI (25), as well as several systematic reviews and observational studies (34-38).

A 2023 meta-analysis found no significant short-term effects of air pollution on lipids, but long-term exposure to PM₁₀, SO₂, and NO₂ was associated with higher TC, TG, LDL-C, and lower HDL-C, albeit with substantial heterogeneity (I² ≥ 69.7%) (34). Another systematic review and meta-analysis, despite limited quantitative evidence, reported significant associations between PM₁₀ and NO₂ and elevated TG levels, with ~80% heterogeneity (35). Similarly, a South Korean prospective cohort of ~13,000 healthy soldiers observed adverse effects of PM₂.₅, NO₂, and O₃ on lipid profiles (39). Despite robust evidence, current lipid management guidelines have yet to incorporate ambient air pollutants as non-dietary risk factors for dyslipidemia (40-44).

### 4.3. Air Pollution and Blood Glucose

National surveys in Iran report a high and rising prevalence of dysglycemia (diabetes or prediabetes), affecting 25.3% of men and 23.6% of women in 2016 (45). Although awareness is relatively high (~80%) and about 70% receive treatment, only 30 - 40% of treated individuals achieve glycemic control (46). In TLGS, exposure to O₃, PM₁₀, and SO₂ was linked to higher risks of IFG, but no pollutant showed a significant association with incident type 2 diabetes mellitus (T2DM). These findings are largely consistent with a 2023 study in Isfahan that linked air pollution exposure to higher FPG, HbA1c, and glucose intolerance (47). However, unlike TLGS, they observed an association between air pollution exposure with increased risk of both diabetes and prediabetes. Furthermore, an Iranian ecologic study also found a significant association between PM₁₀ levels and incident diabetes (23). A population-based retrospective cohort of 73,117 participants in Israel found a modest association between intermediate-term PM₁₀ exposure and higher serum glucose, with the effect twice as strong among individuals with diabetes (38). Supporting this, a 2020 systematic review and meta-analysis reported significant associations of PM₂.₅ and PM₁₀ with incident T2D (48). While the ADA has not yet incorporated air pollution into its clinical guidelines, the association between environmental factors and diabetes risk underscores the importance of considering broader environmental determinants in public health strategies (49).

### 4.4. Air Pollution, Cardiovascular Disease Events, and Mortality

Cardiovascular diseases are among the three leading causes of hospital admissions attributable to air pollution exposure (50). Consistent with TLGS findings, studies from Iran (51-53) and worldwide (54-56)] have shown that exposure to ambient pollutants is significantly associated with higher CVD hospitalization rates. In Arak, each 10 µg/m³ rise in PM₁₀ and NO₂ and each 1 mg/m³ rise in CO were linked to 0.7%, 3.3%, and 9.4% increases in CVD admissions, respectively (57). In Isfahan, a 10-unit AQI increase corresponded to a 7.3% rise in CVD hospitalization (58). Similarly, in Thailand, O₃ was correlated with heart failure (HF) visits, NO₂ with MI admissions, and SO₂ with cerebrovascular events (59).

For CVD mortality, TLGS found that short-term exposure to high AQI values was particularly harmful, leading to an immediate rise in cardiovascular deaths. This aligns with other evidence, including a multi-city case-crossover study showing strong links between NO₂, CO, and PM₂.₅ and cardiovascular mortality, especially in middle-aged and older adults (60). A 2023 national systematic review and meta-analysis also reported PM₁₀ as a major contributor to CVD mortality in Iran (61). Similarly, a Tehran-based study found CVD mortality most strongly associated with PM₁₀, followed by SO₂, NO₂, and O₃, estimating ~5,000 excess all-cause deaths per 100,000 people from these pollutants between 2010 and 2011 (62). Additional Iranian studies further support associations between ambient pollution and respiratory deaths (63), CV mortality (64, 65), hospital admissions (66-69), and cerebrovascular accidents (70). Furthermore, an umbrella review of 56 systematic reviews and meta-analyses provided robust evidence that higher levels of ambient air pollution increase the risk of CVDs and CVD mortality, particularly from stroke and ischemic heart disease (27).

According to the study "Global Effect of Modifiable Risk Factors on Cardiovascular Disease and Mortality" (71), the combined population-attributable fractions of five major traditional cardiovascular risk factors — namely high Body Mass Index (BMI), elevated non-HDL cholesterol, high SBP, type 2 diabetes, and smoking—account for approximately 60% and 30% of 10-year CVD and all-cause mortality, respectively. This suggests that nearly 40% and 70% of CVD and all-cause mortality events occur independently of these factors, thereby highlighting the significance of non-traditional and residual risk factors. Among these, air pollution emerges as an underappreciated contributor, not only exacerbating the burden of traditional risk factors such as high SBP, dyslipidemia, dysglycemia, and obesity, but also serving as a recognized non-traditional risk factor for the onset of CVD itself (50, 72).

### 4.5. Ambient Air Pollutants, Sources, and Mechanisms of Harm

The US Environmental Protection Agency (EPA) reports that ambient air pollutants such as NO₂, SO₂, CO, O₃, and PM₁₀ are primarily emitted through anthropogenic activities, notably from transportation, industrial processes, and combustion of fossil fuels. Nitrogen dioxide is predominantly released from vehicle emissions, power plants, and industrial activities, while SO₂ mainly originates from the burning of coal and oil in power plants and industrial facilities. Carbon monoxide is produced by incomplete combustion of carbon-containing fuels, commonly from motor vehicles and residential heating. Ozone is a secondary pollutant formed through photochemical reactions involving precursor gases like NO₂ and volatile organic compounds under sunlight. Particulate matter consists of inhalable particles with diameters generally 10 micrometers and smaller, arising from both natural sources such as dust storms and anthropogenic sources including construction activities, vehicular emissions, and industrial processes (73).

Ambient NO₂, SO₂, CO, O₃, and PM₁₀ plausibly affect cardiometabolic risk through overlapping pathways that link pulmonary injury to systemic vascular and metabolic dysfunction. Acute exposures trigger airway oxidative stress and inflammation that spill into circulation, disturbing autonomic balance, impairing endothelial function, elevating blood pressure, reducing heart-rate variability, and promoting thrombosis. Chronic exposure sustains low-grade inflammation in vascular, adipose, and hepatic tissues, impairing insulin signaling, worsening dyslipidemia, and driving atherosclerosis and plaque instability. Fine particles may directly translocate into blood and damage vascular or myocardial tissue; O₃ and NO₂ amplify oxidative stress; SO₂ induces bronchoconstriction and autonomic activation; and CO reduces oxygen delivery through carboxyhemoglobin, aggravating myocardial ischemia. Collectively, these mechanisms explain acute surges in ischemic events and heart-failure decompensation after pollution spikes, and long-term increases in HTN, diabetes, and atherosclerotic disease (74).

### 4.6. Clinical Impact and Policy View

The findings from this review, based on studies from the TLGS, highlight the critical role that ambient air pollution plays in shaping the incidence of NCDs and contributing to cardiovascular and metabolic health outcomes. Short-term exposure to pollutants like PM₁₀, SO₂, and CO is consistently linked to elevated risks of HTN, dysglycemia, cardiovascular hospitalization, and increased mortality, particularly during high-pollution periods. Additionally, in the Khajavi et al. (2025) study (15), a correlation of 0.52 was found between SO₂ and CO levels, suggesting a significant relationship between these two pollutants in influencing health risks. These results emphasize the urgent need for targeted public health interventions. Clinically, the results signal that although the harm caused by ambient air pollution is more pronounced among older adults and those with chronic conditions, every individual in this community, regardless of age or health status, is affected by the adverse outcomes of this problem, as demonstrated in the data presented before (25, 47, 64, 75).

In this study, some pollutants appeared to show negative associations with certain cardiometabolic outcomes; however, these are unlikely to represent true biological effects and should be interpreted with caution. Several methodological explanations are plausible. Omitted variable bias, such as unmeasured pollutants (PM₂.₅) or confounders, may have distorted the observed effect. Also, collinearity among pollutants could dilute the harmful impact of some pollutants when correlated with others. Moreover, correlations with unmeasured factors (e.g., socioeconomic status, lifestyle) may further confound the association (76, 77).

This study underscores the urgent need for stricter air quality regulations in urban centers, particularly Tehran, where pollution levels consistently exceed safe thresholds. A key finding is the lag effect; air pollution remains harmful even after concentrations decline, with adverse health effects persisting for days. This exposes the limitations of current short-term policies, such as school or office closures during peak pollution episodes, especially in autumn and winter when inversion worsens air quality. Such temporary approaches cannot offset the lasting health impacts of pollutants. Moreover, our findings show that the risks of air pollution extend beyond high-risk groups — such as the elderly or those with chronic diseases — to the general population. Comprehensive, long-term interventions are urgently required to mitigate these risks and protect public health, including the transition to cleaner fuels, promotion of low- and zero-emission vehicles, wider adoption of effective emission-control technologies, and better alignment of climate investments with health-focused air quality policies.

### 4.7. Conclusions

This scoping review highlights the significant impact of ambient air pollution on cardiovascular and metabolic health in Tehran, driven by both short-term (immediate or lagged) and long-term exposure to pollutants such as PM₁₀, SO₂, and CO. The findings underscore that while older adults and those with chronic conditions are more vulnerable, air pollution adversely affects every individual in the community. Given the consistent evidence from the metropolitan city of Tehran, immediate policy action is required to reduce pollution levels and mitigate its health impacts. Public health initiatives, stricter air quality regulations, and continued research are essential to protect the health of Tehran’s population and reduce the burden of pollution-related diseases.

### 4.8. Strengths, Limitations, and Future Research Directions

This scoping review offers several strengths, providing a comprehensive synthesis of findings from multiple studies conducted within the TLGS. It offers valuable insights into the relationship between ambient air pollution and cardiovascular/metabolic outcomes in Tehran, encompassing a range of pollutants, including PM₁₀, SO₂, NO₂, CO, and O₃. The alignment of the TLGS findings with other national and global studies enhances the generalizability of the results, making them applicable to a broader population.

Several limitations should be acknowledged to guide future research. First, the lack of data on PM₂.₅ — a pollutant with well-established links to cardiovascular and respiratory diseases — limits the ability to fully capture the burden of air pollution. The absence of analyses on multi-pollutant mixtures and source-specific effects further constrains the understanding of combined and differential health risks. Second, spatial variability in air pollution exposure across Tehran was not considered, potentially leading to exposure misclassification and heterogeneous health outcomes across subpopulations. Third, the exclusion of adolescents restricts the assessment of pollution-related health risks in younger populations, who may be particularly vulnerable to adverse effects. Finally, this review focused on cardiometabolic outcomes and did not examine other major conditions associated with air pollution, such as respiratory diseases and cancers, which are critical to comprehensively evaluating its public health impact.

To address these gaps, future research should focus on elucidating the long-term, cumulative effects of chronic exposure to pollutants such as PM₂.₅, NO₂, and CO on the onset and progression of cardiometabolic diseases, as well as other health outcomes linked to air pollution. Large-scale longitudinal studies are essential to capture multi-year exposures, disentangle combined pollutant effects, and assess source-specific contributions from transportation, industry, and residential heating. Equally important is incorporating spatial heterogeneity and examining differential impacts across socio-economic and demographic groups. Finally, evaluating the effectiveness of interventions — including urban planning strategies, regulatory measures, and health promotion programs—will be critical for mitigating risks and enhancing population resilience.
